# Comparing Happiness and Hypomania Risk: A Study of Extraversion and Neuroticism Aspects

**DOI:** 10.1371/journal.pone.0132438

**Published:** 2015-07-10

**Authors:** Tabitha Kirkland, June Gruber, William A. Cunningham

**Affiliations:** 1 Department of Psychology, Bellevue College, Bellevue, Washington, United States of America; 2 Department of Psychology, The Ohio State University, Columbus, Ohio, United States of America; 3 Department of Psychology and Neuroscience, University of Colorado Boulder, Boulder, Colorado, United States of America; 4 Department of Psychology, University of Toronto, Toronto, Ontario, Canada; 5 Department of Marketing, University of Toronto, Toronto, Ontario, Canada; University of Vienna, School of Psychology, AUSTRIA

## Abstract

Positive affect has long been considered a hallmark of subjective happiness. Yet, high levels of positive affect have also been linked with hypomania risk: a set of cognitive, affective, and behavioral characteristics that constitute a dispositional risk for future episodes of hypomania and mania. At a personality level, two powerful predictors of affective experience are extraversion and neuroticism: extraversion has been linked to positive affect, and neuroticism to negative affect. As such, a single personality trait – extraversion – has been linked to both beneficial and harmful outcomes associated with positivity. It is clear that positive affect, in different forms, has divergent consequences for well-being, but previous research has struggled to articulate the nature of these differences. We suggest that the relationship between affect and well-being needs to be situated within the psychological context of the individual – both in terms of more specific forms of extraversion and neuroticism, but also in terms of interactions among personality aspects. Consistent with this idea, we found that two aspects of extraversion (enthusiasm and assertiveness) differentially predicted subjective happiness from hypomania risk and two aspects of neuroticism (volatility and withdrawal) interacted to predict hypomania risk: the highest levels of hypomania risk were associated with the combination of high volatility and low withdrawal. These findings underscore the importance of examining personality at the right level of resolution to understand well-being and dysfunction.

## Introduction

Positive affect is a general dimension of mood reflecting the extent to which people feel subjectively pleasant while being engaged with their environment. High positive affect indicates more subjective pleasure and engagement; low positive affect indicates less subjective pleasure and disengagement. For example, Watson and colleagues [1] suggest that “high positive affect is a state of high energy, full concentration, and pleasurable engagement, whereas low positive affect is characterized by sadness and lethargy” (p. 1963). The temporary experience of positive affect functions as a sign that things are going well, widening the array of thoughts and actions that come to mind [2] and facilitating approach behavior [3].

A wealth of empirical work over the last three decades has demonstrated that the frequent experience of positive affect is an important component of happiness [4,5]. Happiness, also referred to as subjective well-being, is a disposition defined by global positive evaluations of one’s qualities and circumstances. Being a happy person carries many benefits, such as resilience [6], positive social relationships [7], and even longevity [8]. Moreover, happiness seems to cause success and health as much as it reflects these outcomes [9], suggesting a cycle of positivity whereby positive expectations lead to positive experiences, thereby reinforcing those expectations [10]. This work is consistent with the suggestion that positivity is essential to happiness and psychological health.

Yet recently, some research has suggested that positive affect may not always be beneficial [11]. This work has demonstrated that “too much” positive affect–including situationally inappropriate affect–may be linked to substantial psychological and behavioral dysfunction [12], such as hypomania risk: a predisposition for episodes of hypomania and mania. Mania is a core feature of bipolar disorder, a severe and recurrent clinical disorder marked by heightened and persistent disruptions in positive affectivity [13]. Episodes of mania are temporary and occur in a small population, yet personality variables can predict risk for future manic episodes. Hypomania risk is a subclinical analogue of mania and includes a constellation of affective, cognitive, and somatic features that are associated with increased risk for the onset of mania [14]. Hypomania risk, which can be considered a stable, dispositional construct, has also been linked to dysfunctional positive affect. For example, people with hypomania risk report greater positive affect in response to false success feedback [15], as well as elevated levels of positive affect across a variety of daily circumstances [16]. As such, positive affect in mania and hypomania risk has been characterized as “too much of a good thing” [17] in that it reflects intense positivity that is apparently insensitive to context. Taken together, this research suggests that positive affect may be a signal of dysfunction for some individuals.

To understand the divergent implications of positive affect for well-being and mental health, we consider the personality traits that underpin affective experience. Extraversion and neuroticism are powerful predictors of affective experience [18]: extraversion influences positive affect, whereas neuroticism influences negative affect. In this paper, we demonstrate that personality is important to consider when evaluating the seemingly divergent consequences positive affect yields for well-being [19]. Using a taxonomy of personality, we propose that aspects of personality can shape experiences of positive affect into divergent outcomes such that the same general traits (extraversion and neuroticism) have different implications for well-being when examined with greater specificity. We focus in particular on comparing subjective happiness with hypomania risk, a negative outcome that has also been linked with positive affect. We demonstrate how personality aspects differentiate these outcomes.

### Personality Aspects

The most basic way we understand the world is in terms of positive and negative information–reward and threat. Differences in how we respond to reward and threat may originate from differences in our baseline sensitivity. Considerable evidence suggests that extraversion and neuroticism represent the primary personality manifestations of reward and threat sensitivity, respectively [20,21]. Whereas extraversion refers to reward sensitivity and the general tendency to approach, explore, and engage with novelty, neuroticism refers to punishment sensitivity and the general tendency to regulate or restrain potentially disruptive emotions and behaviors [22]. A long tradition of research has examined these structure and functional components of these traits. However, some compelling work has shed new light on the nature of personality by dividing each trait into two distinct aspects [23] based on independent biological and genetic factors [24]. Based on these differences, some researchers have argued that higher-order traits such as extraversion and neuroticism may be best conceptualized as useful heuristic devices rather than discrete psychological structures [24].

According to this revised structure, extraversion is composed of *assertiveness* and *enthusiasm* [23]. Assertiveness reflects an orientation toward agency, drive, and social dominance (e.g., subjective potency for accomplishing goals), whereas enthusiasm denotes friendliness, sociability, and the tendency to experience positive affect [21]. These personality aspects correspond to complementary, yet dissociable, functional strategies for interacting with positive information: motivated approach toward rewards, or “wanting,” versus enjoyment of rewards, or “liking” [25]. “Wanting” refers specifically to incentive salience, a type of motivation that promotes approach toward and consumption of rewards, and is distinguishable from more cognitive forms of desire that involve declarative goals or explicit expectations of future outcomes. By contrast, “liking” refers specifically to the positive hedonic impact of a reward, even in the absence of conscious awareness [26]. Because these strategies are also rooted in separate neurochemical systems (dopamine vs. opioids) [27], they may at times become decoupled. This may help to explain diverse life outcomes associated with extraversion–which has been associated with positive behaviors, such as the sociability and excitement found in people who self-identify as happy, as well as with negative behaviors, such as poor impulse control and hypomania risk [28], a dispositional predisposition to episodes of (hypo)mania [14].

Neuroticism differentiates into *volatility* and *withdrawal* [23]. Volatility refers to emotional instability, difficulty controlling emotional impulses, and susceptibility to negative affect directed outward (disinhibition), whereas withdrawal refers to susceptibility to negative affect directed inward (inhibition). These personality aspects serve related yet distinct functional roles for dealing with negative information. Volatility serves to orient the individual vigilantly toward negativity: when faced with a perceived threat, volatile individuals often choose to reduce the threat by confronting it directly. On the other hand, withdrawal serves to promote a passive avoidance strategy such that the individual reduces his or her chances of entering a situation in which threats could occur. Although these aspects can work together, they also may at times diverge. For example, individuals with hypomania risk are often volatile, demonstrating irritability and emotional instability [14], yet are not necessarily prone to withdrawal; rather, they often demonstrate a maladaptive tendency to approach all stimuli regardless of their potential for benefit or harm [29].

### Research Aims

**Fig 1:**
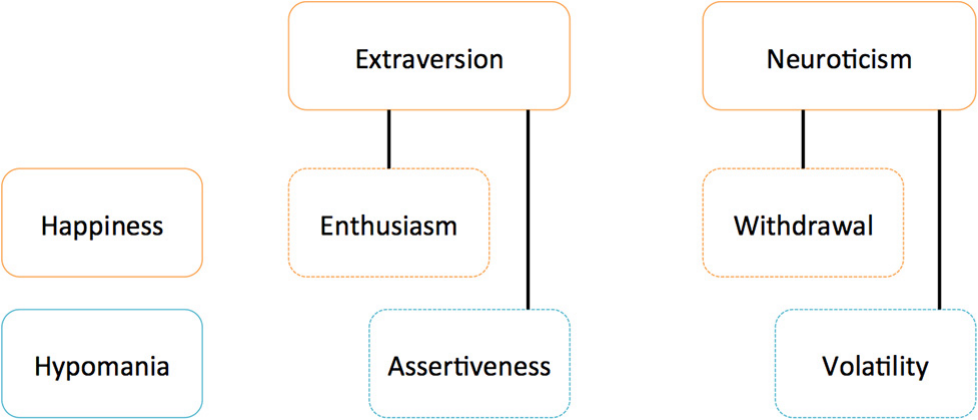
Conceptual Links Among Aspects of Extraversion and Neuroticism, Subjective Happiness, and Hypomania Risk. Enthusiasm (extraversion) and withdrawal (neuroticism) are most closely linked to subjective happiness; assertiveness (extraversion) and volatility (neuroticism) are most closely linked to hypomania risk.

Because aspects of extraversion and neuroticism correspond to different functional strategies for dealing with reward and threat, we sought to discover whether examining these aspects would help to make sense of the diverse life outcomes associated with positive affect. The present work tests whether and how aspects of extraversion and neuroticism differentiate subjective happiness from hypomania risk, dispositions that are both associated with positive affect but which have divergent consequences for well-being. If personality aspects play an important role in differentiating functional outcomes, there should be different patterns in the prevalence of these aspects between subjective happiness and hypomania risk. The conceptual relationships described below are illustrated in **Fig 1**.

Aim 1: Aspects of extraversion differentiate subjective happiness and hypomania risk. We examined the possibility that although the broader construct of extraversion would be associated with both subjective happiness and hypomania risk, aspects of extraversion would differentiate between them. Specifically, assertiveness (“wanting”) without enthusiasm (“liking”) would be more likely to predict hypomania risk, whereas enthusiasm alone would be more likely to predict subjective happiness.

Aim 2: Aspects of neuroticism differentiate subjective happiness and hypomania risk. We examined the possibility that the aspects of neuroticism–volatility and withdrawal–would diverge for hypomania risk such that hypomania risk would be associated with high volatility and low withdrawal. Subjective happiness, by contrast, would be associated with lower overall neuroticism.

## Method

### Data Collection and Aggregation

We collected data across three waves. The differences between the waves were that our second and third waves included some additional clinical measures and were conducted online using a U.S. community sample rather than college undergraduates. To maximize power, we combined these three studies into a single data set for analyses. Our decision to collect data in multiple waves was based on the recent emphasis on replication of studies in psychology [30]. This decision was not made *a priori*. We initially conducted a single study, then decided to conduct a second one with additional measures to ensure our findings held controlling for clinical symptoms. After collecting our second wave of data, we decided to collect one more wave to make sure the results replicated. Sample sizes were based on the size of the available volunteer research pool (Wave 1) and available funds for paid participants (Waves 2, 3). We chose to analyze all waves together following the recommendations of Schimmack [31], who suggests that analyzing multiple replications of a study within a single statistical model increases the total power across studies and contributes to the credibility of findings. All raw data can be found in S1 File.

### Participants and Design

Our first wave of data collection included 352 undergraduates (61% female; age *M* = 19.29, range = 18–45) who completed questionnaires online in exchange for course credit. Our second wave of data collection included 471 U.S. community participants who were recruited through Mechanical Turk, a paid survey website run by Amazon.com that offers a demographically diverse sample and generally reliable data [32]. Participants who incorrectly answered two catch questions designed to ensure attention were removed, leaving a sample of 466; however, seven participants (1.5% of the sample) failed to complete all measures, so analyses include data from only the 459 participants who completed all questions (62% female, age *M* = 33.06, range = 18–81). Our third wave of data collection included 201 U.S. community participants who were recruited through Mechanical Turk. All participants passed the attention check, but 23 people (11.4% of the sample) failed to complete all measures, so analyses include data from only the 178 participants who completed all questions (58% female, age *M* = 34.5, *SD* = 13.73, range = 18–82). Across three waves of data collection, then, our total sample size for analysis was 989 participants. Results did not differ when dropped participants were included in analyses. See S1 Table for a summary of analyses including these individuals. We conducted a *post hoc* power analysis to determine the effect sizes that could be detected in our study. Our study had high statistical power: all correlations of *r* = .08 (*R* ^2^ = .006) or stronger met or exceeded Cohen’s [33] benchmark of .80 for power.

### Ethics Statement

This research was approved by the Ohio State University Institutional Review Board and included written informed consent for all participants.

### Measures

The following dispositional measures were included in all waves of data collection (i.e., participants reported on how they usually tended to think, feel, and act). Trait positive affect (PA) and negative affect (NA) were measured with the Positive Activation and Negative Activation Schedule [1,34], a 20-item scale that measures average levels of activated (i.e., aroused) PA and NA. Participants rate the extent to which they tend to feel each of several affective states “generally … that is, how you feel on average,” including items such as “irritable” and “enthusiastic.” Trait affect is conceptually distinct from state (temporary) affect: whereas state affect refers only to one’s current mood, and can be influenced by a variety of contextual factors, trait affect refers to how often people generally tend to experience a variety of states. As such, trait PA and NA are more stable than state PA and NA and can be considered dispositional measures of affect.

Extraversion and neuroticism were measured using the Big Five Aspects Scale [23], a 100-item scale that also assesses other personality traits and was used to measure separable aspects of extraversion and neuroticism. Extraversion and neuroticism (and their aspects) are considered stable, dispositional constructs that represent the personality variables governing our global responses to reward and threat.

Subjective happiness (abbreviated hereafter as “happiness”) was measured with the Subjective Happiness Scale (SHS) [35], a four-item scale that measures self-reported trait subjective happiness. Participants rate themselves on a 1–7 semantic differential scale for each question, with labels only at the endpoints. A sample item is: “In general, I consider myself: (1) not a very happy person–(7) a very happy person.”

Hypomania risk was measured with the Hypomanic Personality Scale (HPS) [14], a self-report questionnaire with high internal consistency (α = .89 in the present study) and predictive validity for the onset of manic episodes. The HPS consists of 48 true-false items capturing episodic shifts in emotion (“I often feel excited and happy for no apparent reason”), behavior (“there are often times when I am so restless that it is impossible for me to sit still”) and energy (“I very frequently get into moods where I wish I could be everywhere and do everything at once”). Several studies suggest that elevated scores on the HPS generalize to clinical samples of those diagnosed with bipolar disorder. First, previous research indicates that high scores on the HPS correlate with clinical diagnoses of bipolar disorder [14] and current mania symptoms [36]. In these studies, 78% of undergraduates who scored above the high-risk cutoff reported experiencing hypomanic episodes, and 25% actually qualified for a DSM-IV diagnosis of bipolar disorder [14]. Second, in one study, participants who scored above the high-risk threshold were found to have an increased risk for the development of manic episodes (25% compared to 10%) at a 13-year follow up assessment [37].

To evaluate possible conceptual overlap between our measures of happiness and hypomania risk, we review research focused on the structure of each scale. Such work suggests that the SHS is a one-dimensional measure of happiness [35], focusing on an individual’s global self-evaluation of their happiness. In comparison to other measures or models of happiness, the SHS intentionally does not make a distinction between affective and cognitive components [38,39]. By contrast, the HPS is a multidimensional measure composed of a variety of latent constructs. For example, the initial report of the HPS included a principal components analysis with 12 factors with eigenvalues greater than one; however, only two factors seemed to fit the items: fifteen items loaded onto one component reflecting hypomanic symptoms (e.g., hyperactivity, mood fluctuations), and six items loaded onto another component reflecting exhibitionism [14]. The remaining factors did not account for much additional variance. More recently, multidimensional scaling analyses of the HPS have revealed three subscales: social vitality, mood volatility, and excitement [40]. Social vitality and excitement are related to extraversion, and happiness is also related to extraversion; indeed, extraversion is considered to be the primary personality manifestation of high positive affect, supporting the importance of comparing positive affect between happiness and hypomania risk.

Although happiness and hypomania risk are related (i.e., self-reported feelings of happiness may be present among people with hypomania risk), they appear to be distinct constructs that, in our data, are at best weakly correlated, *r*s = .07, .11, .02 (Waves 1, 2, and 3, respectively). Happiness, like hypomania risk, is a broader construct than social vitality and excitement; it may include these concepts, but does not exhibit a one-to-one mapping with them. For example, social vitality items on the HPS include “I am so good at controlling others that it sometimes scares me” and “I seem to have an uncommon ability to persuade and inspire others,” many of which seem to reflect attention-seeking and social manipulation. Excitement items on the HPS include “I am frequently in such high spirits that I can’t concentrate on any one thing for too long” and “I often have moods where I feel so energetic and optimistic that I feel I could outperform almost anyone at anything.” These items reflect high arousal positive affective states that are not necessarily functional. By contrast, the SHS measures a person’s global self-evaluation as a happy or unhappy person. This leaves the idea of what constitutes a “happy person” up to the individual. Although individuals vary widely in the sources of their personal happiness, most people have an intuitive knowledge of what happiness means for them and whether or not they have achieved it [41]. These global self-evaluations on the SHS are meaningfully different from the moods, attitudes, and behaviors on the HPS.

Given the goals of this work (i.e., comparing overall trait happiness to overall hypomania risk), we did not judge it appropriate to analyze the HPS subscales separately in our main analyses. However, we include a secondary analysis section at the end of our Results in which we assess the degree of overlap between each HPS subscale and our personality measures of interest.

Waves 2 and 3 also included measures of potential mood symptom confounds: mania symptoms, measured with the Altman Self-Rating Mania Scale (ASRM) [42], which consists of five items assessing cognitive, affective, and somatic symptoms of mania over the last two weeks, and depression symptoms, measured with the Beck Depression Inventory-Short Form (BDI) [43], which consists of 13 items assessing cognitive, affective, and somatic symptoms of depression over the last two weeks. Both symptoms of mania and depression commonly co-occur with experiences of hypomania risk, so we controlled for these variables in secondary analyses to ensure our findings held independent of these clinical states [17]. This control seems especially relevant given the findings of a recent paper examining hypomania risk among patients with mood disorders, which suggests that the HPS may be a measure of both personality style and current hypomanic/manic mood symptoms [44].

Table 1 reports descriptive statistics across three waves of data collection, and Tables 2, 3, and 4 report the individual descriptive statistics for each wave.

**Table 1:** Correlation matrix of measured variables with internal consistency, means, standard deviations, and ranges (all waves).

	PA	NA	SHS	HPS	E	EA	EE	N	NV	NW	ASRM	BDI-SF
**PA**	(0.90)											
**NA**	-0.12	(0.91)										
**SHS**	0.57***	-0.42***	(0.88)									
**HPS**	0.24***	0.28***	0.11***	(0.89)								
**Extraversion**	0.55***	-0.28***	0.56***	0.34***	(0.89)							
** Assertiveness**	0.44***	-0.19***	0.34***	0.38***	0.85***	(0.86)						
** Enthusiasm**	0.50***	-0.29***	0.61***	0.19***	0.86***	0.46***	(0.87)					
**Neuroticism**	-0.37***	0.62***	-0.58***	0.13***	-0.40***	-0.30***	-0.38***	(0.91)				
** Volatility**	-0.27***	0.54***	-0.45***	0.22***	-0.23***	-0.11***	-0.28***	0.91***	(0.89)			
** Withdrawal**	-0.41***	0.59***	-0.60***	0.01	-0.50***	-0.44***	-0.41***	0.90***	0.64***	(0.86)		
**ASRM**	0.34***	0.09*	0.25***	0.47***	0.26***	0.23***	0.21***	-0.07	-0.02	-0.11***	(0.77)	
**BDI-SF**	-0.39***	0.60***	-0.55***	0.15***	-0.38***	-0.26***	-0.40***	0.60***	0.50***	0.61***	-0.04	(0.89)
**Mean**	3.20	2.01	4.69	18.53	3.37	3.30	3.44	2.79	2.75	2.84	4.05	9.66
**SD**	0.78	0.80	1.36	9.27	0.58	0.68	0.69	0.68	0.77	0.74	3.72	9.28
**Range**	1–5	1–5	1–7	0–47	1.1–5	1.1–5	1–5	1–5	1–5	1–5	0–19	0–39

* *p* < 05

** *p* < .01

*** *p* < .001

PA = positive affect and NA = negative affect (Positive and Negative Affect Schedule); SHS = Subjective Happiness Scale; SWLS = Satisfaction with Life Scale; HPS = Hypomanic Personality Scale. Extraversion (E) and neuroticism (N) were measured with the Big Five Aspects Scale: assertiveness (EA) and enthusiasm (EE) are aspects of extraversion; volatility (NV) and withdrawal (NW) are aspects of neuroticism. ASRM = Altman Self-Rating Mania Scale; BDI = Beck Depression Inventory-Short Form. Numbers on the main diagonal denote Cronbach's alphas as measures of internal consistency.

**Table 2:** Correlation matrix of measured variables with internal consistency, means, standard deviations, and ranges (Wave 1).

	PA	NA	SHS	HPS	E	EA	EE	N	NV	NW
**PA**	(.86)									
**NA**	-0.18***	(.86)								
**SHS**	0.58***	-0.42***	(.84)							
**HPS**	0.06	0.23***	0.07	(.87)						
**Extraversion**	0.53***	-0.35***	0.53***	0.21***	(.87)					
** Assertiveness**	0.41***	-0.24***	0.32***	0.3***	0.84***	(.81)				
** Enthusiasm**	0.49***	-0.36***	0.59***	0.07	0.86***	0.46***	(.83)			
**Neuroticism**	-0.33***	0.56***	-0.49***	0.16**	-0.39***	-0.27***	-0.4***	(.84)		
** Volatility**	-0.21***	0.45***	-0.33***	0.27***	-0.2***	-0.04	-0.28***	0.87***	(.81)	
** Withdrawal**	-0.38***	0.52***	-0.51***	0	-0.49***	-0.44***	-0.4***	0.85***	0.48***	(.75)
**Mean**	3.44	2.21	5	20.75	3.46	3.34	3.57	2.82	2.78	2.86
**SD**	0.61	0.65	1.21	8.62	0.51	0.58	0.62	0.52	0.63	0.58
**Range**	1–5	1–4	1–7	1–40	1.8–5	1.4–5	1.8–5	1.05–4.25	1–4.5	1–4.4

* *p* < .05

** *p* < .01

*** *p* < .001

PA = positive affect and NA = negative affect (Positive and Negative Affect Schedule); SHS = Subjective Happiness Scale; SWLS = Satisfaction with Life Scale; HPS = Hypomanic Personality Scale. Extraversion (E) and neuroticism (N) were measured with the Big Five Aspects Scale: assertiveness (EA) and enthusiasm (EE) are aspects of extraversion; volatility (NV) and withdrawal (NW) are aspects of neuroticism. ASRM = Altman Self-Rating Mania Scale; BDI = Beck Depression Inventory-Short Form. Numbers on the main diagonal denote Cronbach's alphas as measures of internal consistency.

**Table 3:** Correlation matrix of measured variables with internal consistency, means, standard deviations, and ranges (Wave 2).

	PA	NA	SHS	HPS	E	EA	EE	N	NV	NW	ASRM	BDI-SF
**PA**	(.91)											
**NA**	-0.20***	(.91)										
**SHS**	0.55***	-0.5***	(.91)									
**HPS**	0.23***	0.22***	0.11*	(.90)								
**Extraversion**	0.56***	-0.32***	0.57***	0.39***	(.90)							
** Assertiveness**	0.46***	-0.23***	0.36***	0.44***	0.85***	(.88)						
** Enthusiasm**	0.49***	-0.31***	0.61***	0.23***	0.85***	0.45***	(.87)					
**Neuroticism**	-0.43***	0.66***	-0.64***	0.06	-0.42***	-0.32***	-0.4***	(.94)				
** Volatility**	-0.33***	0.57***	-0.52***	0.15***	-0.27***	-0.14**	-0.31***	0.92***	(.92)			
** Withdrawal**	-0.47***	0.64***	-0.65***	-0.05	-0.51***	-0.45***	-0.42***	0.91***	0.69***	(.89)		
**ASRM**	0.32***	0.06	0.26***	0.45***	0.26***	0.22***	0.22***	-0.08	-0.02	-0.12**	(.78)	
**BDI-SF**	-0.4***	0.59***	-0.55***	0.11*	-0.41***	-0.29***	-0.41***	0.61***	0.51***	0.61***	-0.09	(.88)
**Mean**	3.08	1.91	4.58	17.29	3.35	3.31	3.39	2.76	2.72	2.8	3.95	9.59
**SD**	0.84	0.82	1.40	9.26	0.62	0.73	0.72	0.77	0.86	0.82	3.75	9.11
**Range**	1–5	1–4.8	1–7	0–43	1.1–4.9	1.2–5	1–5	1–5	1–5	1–5	0–19	0–39

* *p* < .05

** *p* < .01

*** *p* < .001

PA = positive affect and NA = negative affect (Positive and Negative Affect Schedule); SHS = Subjective Happiness Scale; SWLS = Satisfaction with Life Scale; HPS = Hypomanic Personality Scale. Extraversion (E) and neuroticism (N) were measured with the Big Five Aspects Scale: assertiveness (EA) and enthusiasm (EE) are aspects of extraversion; volatility (NV) and withdrawal (NW) are aspects of neuroticism. ASRM = Altman Self-Rating Mania Scale; BDI = Beck Depression Inventory-Short Form. Numbers on the main diagonal denote Cronbach's alphas as measures of internal consistency.

**Table 4:** Correlation matrix of measured variables with internal consistency, means, standard deviations, and ranges (Wave 3).

	PA	NA	SHS	HPS	E	EA	EE	N	NV	NW	ASRM	BDI-SF
**PA**	(.91)											
**NA**	-0.08	(.91)										
**SHS**	0.53***	-0.45***	(.91)									
**HPS**	0.33***	0.39***	0.02	(.90)								
**Extraversion**	0.54***	-0.24***	0.51***	0.32***	(.90)							
** Assertiveness**	0.41***	-0.09	0.28***	0.37***	0.86***	(.88)						
** Enthusiasm**	0.52***	-0.33***	0.59***	0.18*	0.85***	0.47***	(.87)					
**Neuroticism**	-0.32***	0.63***	-0.62***	0.26***	-0.37***	-0.28***	-0.36***	(.94)				
** Volatility**	-0.22***	0.59***	-0.49***	0.33***	-0.19**	-0.10	-0.24**	0.91***	(.92)			
** Withdrawal**	-0.38***	0.57***	-0.66***	0.16*	-0.49***	-0.40***	-0.43***	0.92***	0.69***	(.89)		
**ASRM**	0.40***	0.17*	0.27***	0.50***	0.25***	0.26***	0.18*	-0.08	-0.03	-0.12	(.78)	
**BDI-SF**	-0.36***	0.62***	-0.56***	0.23**	-0.31***	-0.15*	-0.39***	0.58***	0.48***	0.59***	0.07	(.88)
**Mean**	3.04	1.90	4.33	17.57	3.27	3.20	3.33	2.84	2.77	2.9	4.26	9.89
**SD**	0.82	0.91	1.40	9.71	0.59	0.70	0.69	0.71	0.75	0.80	3.62	9.71
**Range**	1.1–4.9	1–5	1–7	0–47	1.3–5	1.1–5	1.4–5	1.15–4.7	1.1–4.8	1.1–5	0–15	0–37

* *p* < .05

** *p* < .01

*** *p* < .001

PA = positive affect and NA = negative affect (Positive and Negative Affect Schedule); SHS = Subjective Happiness Scale; SWLS = Satisfaction with Life Scale; HPS = Hypomanic Personality Scale. Extraversion (E) and neuroticism (N) were measured with the Big Five Aspects Scale: assertiveness (EA) and enthusiasm (EE) are aspects of extraversion; volatility (NV) and withdrawal (NW) are aspects of neuroticism. ASRM = Altman Self-Rating Mania Scale; BDI = Beck Depression Inventory-Short Form. Numbers on the main diagonal denote Cronbach's alphas as measures of internal consistency.

### Data Analyses

We constructed a series of four general linear model (GLM) analyses with subjective happiness and hypomania risk as outcome variables. The first two models examined the relationships of happiness and hypomania risk with affect (PA and NA) and personality (extraversion and neuroticism). The third model predicted happiness and hypomania risk from aspects of extraversion (assertiveness, enthusiasm) and neuroticism (volatility, withdrawal), as well as the interactions between each trait-related aspect. Finally, in a fourth model, we demonstrate that these relationships with aspects of extraversion and neuroticism hold even when controlling for PA and NA. In a secondary set of analyses, we controlled for current symptoms of depression (BDI) or mania (ASRM)–both of which can be present in bipolar disorder–to rule out the hypothesis that these symptoms contributed to differences between happiness and hypomania risk. A secondary set of analyses use GLMs to assess the degree of overlap between each HPS subscale and our personality measures of interest. All variables remained unstandardized. Table 5 presents *t*-tests and *p*-values for each analysis.

**Table 5:** *T*-values for all analyses.

Analysis	Predictors	SHS	HPS	HPS2
**Analysis 1**	PA	22.01***	9.3***	5.47***
	NA	-14.85***	10.74***	4.63***
	ASRM			10.72***
	BDI			2.94***
**Analysis 2**	Extraversion	15.22***	14.86***	12.17***
	Neuroticism	-16.81***	10.05***	4.7***
	ASRM			11.81***
	BDI			5.48***
**Analysis 3**	Assertiveness	0.06	0.23	0.27
	Enthusiasm	5.28***	-1.99*	-1.58
	Assertiveness x Enthusiasm	-0.53	3.04**	2.57*
	Volatility	1.66	4.57***	1.9
	Withdrawal	-2.69**	2.48*	0.09
	Volatility x Withdrawal	-3.05**	-2.09*	-0.13
	ASRM			11.67***
	BDI			5.08***
**Analysis 4**	Assertiveness	-0.66	-0.12	0.13
	Enthusiasm	4.29***	-2.13*	-1.6
	Assertiveness x Enthusiasm	-0.43	3.22**	2.62**
	Volatility	1.74	3.93***	1.97*
	Withdrawal	-1.86	1.13	-0.09
	Volatility x Withdrawal	-2.71**	-2.22*	-0.61
	PA	11.16***	2.61**	1.19
	NA	-3.79***	9.75***	4.95
	ASRM			10.05***
	BDI			3.48***

* *p* < .05

** *p* < .01

*** *p* < .001

PA = positive affect and NA = negative affect (Positive and Negative Affect Schedule); ASRM = Altman Self-Rating Mania Scale; BDI = Beck Depression Inventory-Short Form; SHS = Subjective Happiness Scale; HPS = Hypomanic Personality Scale. The column titled 'HPS2' represents results from a secondary set of analyses that include current symptoms of mania (ASRM) and depression (BDI), with HPS as the outcome variable.

## Results

### Relationships among Affect, Personality Aspects, Happiness, and Hypomania Risk

Our first set of analyses sought to confirm the relationships among PA and NA with happiness and hypomania risk. We predicted that happiness would be associated with high PA and low NA, whereas hypomania risk would be associated with high PA and high NA. We conducted two GLMs predicting happiness and hypomania risk from PA and NA. Consistent with previous work, happiness was associated with high PA, *β*_*HAP*_ = .51, and low NA, *β*_*HAP*_ = -.35, whereas hypomania risk was associated with high PA, *β*_*HYP*_ = .27, and high NA, *β*_*HYP*_ = .32. Controlling for current symptoms of mania and depression, results for hypomania risk remained consistent, *β*_*HYP*_ = .31 (PA) and *β*_*HYP*_ = .19 (NA). In other words, both outcomes–happiness and hypomania risk–were consistent with PA, but diverged when it came to NA.

Our second set of analyses sought to confirm the relationship of personality traits–specifically, extraversion and neuroticism–with happiness and hypomania risk. We predicted that happiness would be positively related to extraversion and negatively related to neuroticism, whereas hypomania risk would be positively related to extraversion and positively related to neuroticism. We conducted two GLMs predicting happiness and hypomania risk from extraversion and neuroticism. Consistent with previous work, happiness was associated with high extraversion, *β*_*HAP*_ = .39, and low neuroticism, *β*_*HAP*_ = -.43, whereas hypomania risk was associated with high extraversion, *β*_*HYP*_ = .46, and high neuroticism, *β*_*HYP*_ = .31. Controlling for current symptoms of mania and depression, results for hypomania risk remained consistent, *β*_*HYP*_ = .43 (extraversion) and *β*_*HYP*_ = .18 (neuroticism). In other words, both outcomes–happiness and hypomania risk–were consistent for extraversion, but diverged for neuroticism.

**Fig 2:**
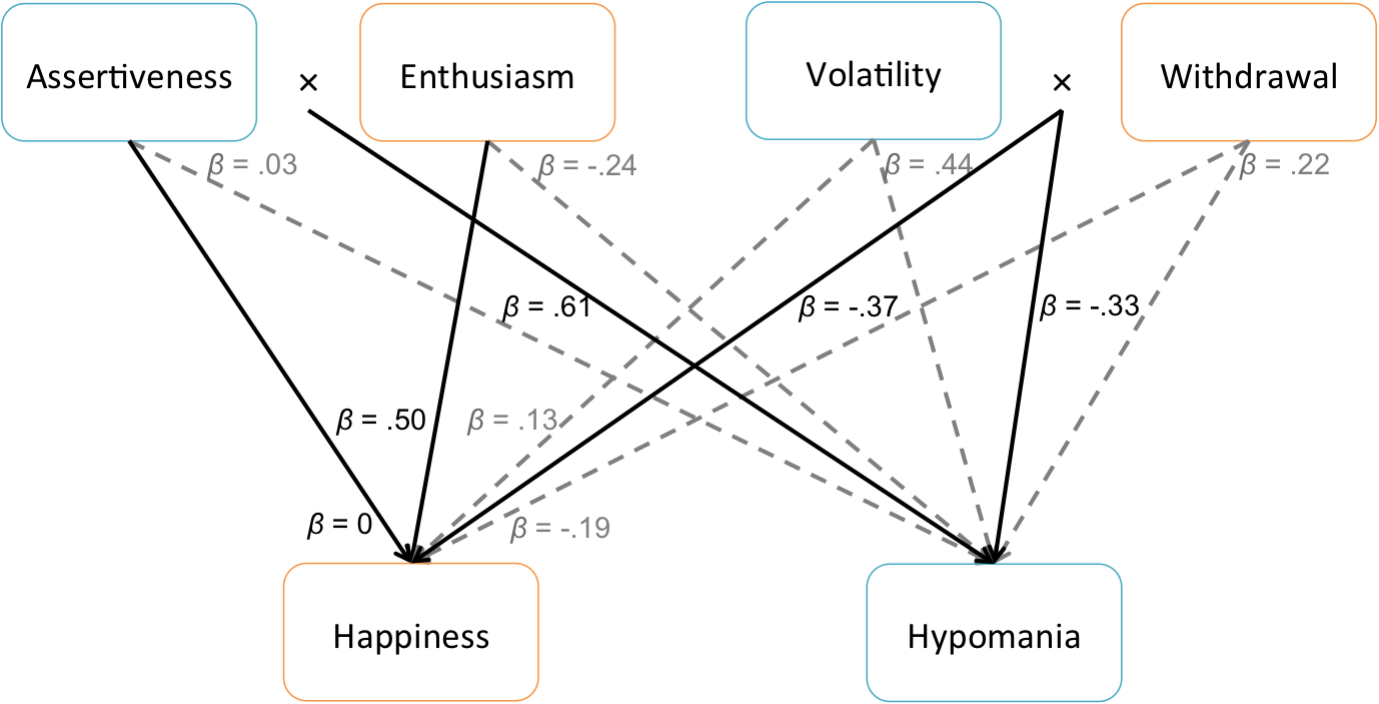
Relationships Among Aspects of Extraversion and Neuroticism, Subjective Happiness, and Hypomania Risk. Standardized beta values are provided as measures of effect size. Main effects qualified by interactions are presented in light grey with dashed lines. Main effects not qualified by interactions, and significant interactions, are presented in black with solid lines. Colored outlines further link personality aspects to the disposition (happiness/orange or hypomania risk/blue) with which they are most closely associated, based on interaction analyses.

**Fig 3:**
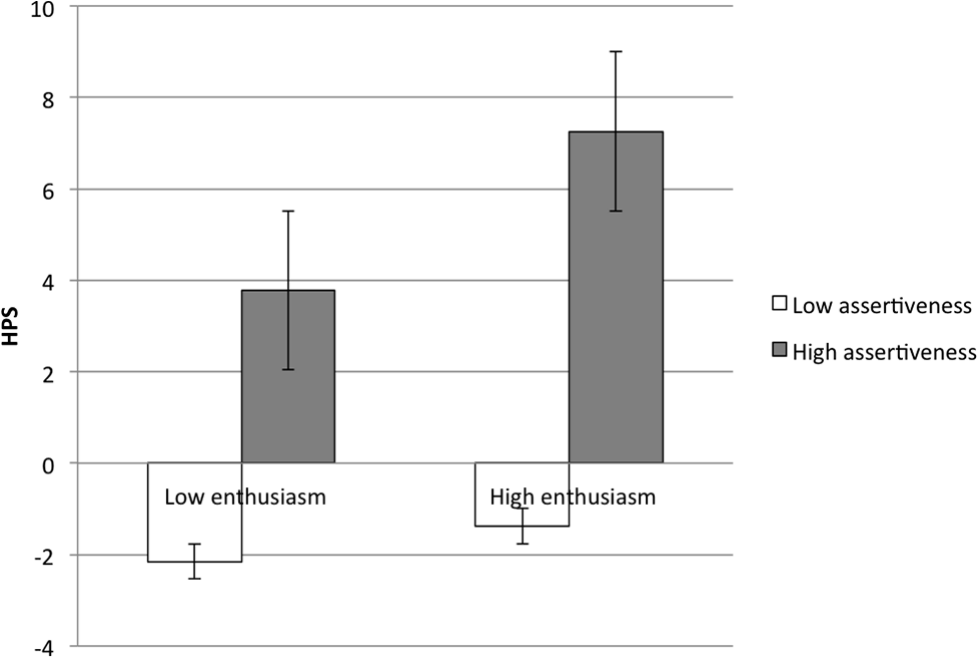
Interaction of Assertiveness and Enthusiasm Predicts Hypomania Risk. HPS = Hypomanic Personality Scale.

**Fig 4:**
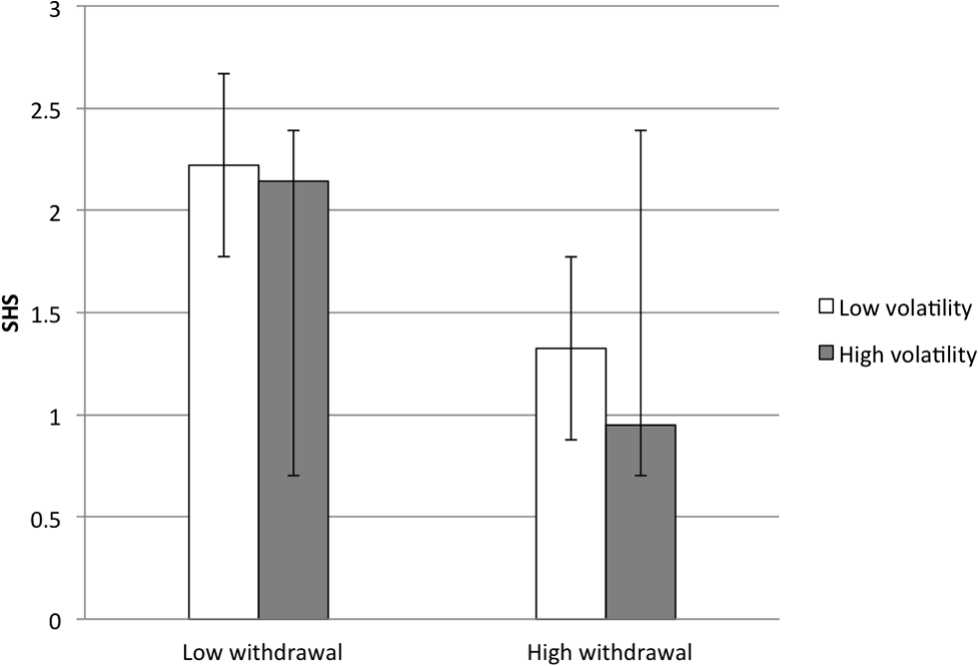
Interaction of Volatility and Withdrawal Predicts Subjective Happiness. SHS = Subjective Happiness Scale.

**Fig 5:**
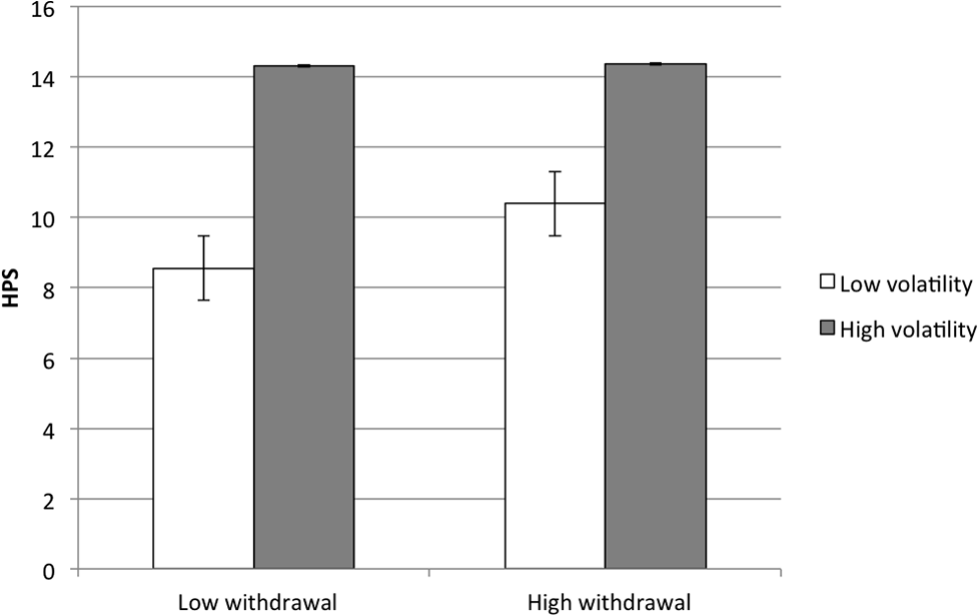
Interaction of Volatility and Withdrawal Predicts Hypomania Risk. HPS = Hypomanic Personality Scale.

Our third set of analyses were designed to examine the possibility that aspects of extraversion and neuroticism would diverge for happiness and hypomania risk. Within extraversion, we predicted that hypomania risk would be associated with assertiveness, whereas happiness would be associated with enthusiasm. Within neuroticism, we predicted that hypomania risk would be associated with high volatility and low withdrawal, whereas happiness would be associated with lower overall neuroticism. We conducted two GLMs predicting happiness and hypomania risk from aspects of extraversion (assertiveness, enthusiasm, and their interaction) and neuroticism (volatility, withdrawal, and their interaction). We found not only that these aspects provided greater nuance in predicting happiness and hypomania risk, but also that interactions among trait-related aspects suggested particular combinations of personality traits for whom happiness, or hypomania risk, would be most likely. **Fig 2** illustrates the main-effect relationships among these constructs, and **Figs 3–5** illustrate the interactions.

When examining aspects of extraversion, happiness was not associated with assertiveness, *β*_*HAP*_ = 0, and was positively associated with enthusiasm, *β*_*HAP*_ = .50. There was no significant interaction between these aspects. By contrast, hypomania risk was not associated with assertiveness, *β*_*HYP*_ = .02, and negatively associated with enthusiasm, *β*_*HYP*_ = -.24; however, these relationships among assertiveness, enthusiasm, and hypomania risk were qualified by a significant interaction, *β*_*HYP*_ = .61, indicating that higher levels of assertiveness were always associated with hypomania risk, even if enthusiasm was low (**Fig 3**). The highest levels of hypomania risk were endorsed among people with both high assertiveness and high enthusiasm, and the lowest levels were endorsed among people with low assertiveness, regardless of enthusiasm. This interaction suggests that assertiveness is the aspect of extraversion that most influences hypomania risk. The non-significant main effect of assertiveness on hypomania risk can be explained as follows: Assertiveness was significantly predicted by each of the aspects of neuroticism, *βs* = .56 (volatility) and .43 (withdrawal), as well as their interaction, *β* = -.48. Because of this, when predicting hypomania risk from all personality variables simultaneously, the neuroticism interaction accounted for the main effect of assertiveness–but not the assertiveness/enthusiasm interaction. Further confirming this, when excluding the volatility/withdrawal interaction from the model, assertiveness significantly predicted hypomania risk, *β* = .61. Controlling for current symptoms of mania and depression, relationships among assertiveness, enthusiasm, and hypomania risk remained consistent, *β*_*HYP*_ = .04 (assertiveness), *β*_*HYP*_ = -.20 (enthusiasm), and *β*_*HYP*_ = .55 (assertiveness × enthusiasm).

When examining aspects of neuroticism, happiness was not associated with volatility, *β*_*HAP*_ = .13, and negatively associated with withdrawal, *β*_*HAP*_ = -.19. These relationships among volatility, withdrawal, and happiness were qualified by a significant interaction, *β*_*HAP*_ = -.37, indicating that volatility had more of a negative impact on happiness at high levels of withdrawal compared to low levels: the highest levels of happiness were endorsed among people with low withdrawal, regardless of volatility, and the lowest levels of happiness were endorsed among people with high withdrawal and high volatility (**Fig 4**). This interaction suggests that withdrawal is the aspect of neuroticism that most influences happiness. By contrast, hypomania risk was associated with high volatility, *β*_*HYP*_ = .44, and high withdrawal, *β*_*HYP*_ = .22; however, these relationships among volatility, withdrawal, and hypomania risk were also qualified by a significant interaction, *β*_*HYP*_ = -.33, indicating that the highest levels of hypomania risk were endorsed among people with high volatility, regardless of withdrawal, whereas the lowest levels of hypomania risk were endorsed among people with both low volatility and low withdrawal (**Fig 5**). This interaction suggests that volatility is the aspect of neuroticism that most influences hypomania risk. Controlling for current symptoms of mania and depression, the relationship between volatility and hypomania risk remained consistent, *β*_*HYP*_ = .20 (volatility), but withdrawal was no longer a significant predictor, *β*_*HYP*_ = 0 (withdrawal), *β*_*HYP*_ = -.02 (volatility × withdrawal). In summary, our data indicate that subjectively happy people are most likely to be highly enthusiastic and not withdrawn, whereas people with a hypomania risk are mostly likely to be highly assertive and volatile.

Finally, to understand the extent to which personality aspects predict happiness and hypomania risk above and beyond the influence of affect, the fourth model predicted happiness and hypomania risk from aspects of extraversion and neuroticism while controlling for PA and NA. Within aspects of extraversion, happiness remained unrelated to assertiveness, *β*_*HAP*_ = -.06, and positively associated with enthusiasm, *β*_*HAP*_ = .38, with no significant interaction. Hypomania risk remained unrelated to assertiveness, *β*_*HYP*_ = -.01, and negatively related to enthusiasm, *β*_*HYP*_ = -.24; the assertiveness × enthusiasm interaction remained significant, *B*_*HYP*_ = .61, and its interpretation was consistent with the third set of analyses: the highest levels of hypomania risk were endorsed among people with high enthusiasm and high assertiveness, and the lowest levels among people with low assertiveness, regardless of enthusiasm. Controlling for current symptoms of mania and depression, relationships among assertiveness, enthusiasm, and hypomania risk remained consistent, *β*_*HYP*_ = .02 (assertiveness), *β*_*HYP*_ = -.20 (enthusiasm), and *β*_*HYP*_ = .55 (assertiveness × enthusiasm).

Within aspects of neuroticism, happiness remained unrelated to volatility, *β*_*HAP*_ = .13, and was marginally (*p* = .06) negatively related to withdrawal, *β*_*HAP*_ = -.13; the volatility × withdrawal interaction remained significant, *β*_*HAP*_ = -.31, and its interpretation remained consistent with the third set of analyses: the highest levels of happiness were endorsed among people with low withdrawal, regardless of volatility, and the lowest levels of happiness were endorsed among people with high withdrawal and high volatility. Hypomania risk remained associated with high volatility, *β*_*HYP*_ = .36, but was not associated with withdrawal, *β*_*HYP*_ = .10; however, the volatility × withdrawal interaction remained significant, *β*_*HYP*_ = -.33. In contrast to the third set of analyses, which did not control for affect, this interaction showed that the highest levels of hypomania risk were endorsed among people with the unique combination of high volatility and low withdrawal, strengthening our suggestion that volatility is the aspect of neuroticism that most influences hypomania risk. Controlling for current symptoms of mania and depression, relationships among volatility, withdrawal, and hypomania risk remained consistent, *β*_*HYP*_ = .20 (volatility), *β*_*HYP*_ = 0 (withdrawal), but the interaction was no longer significant, *β*_*HYP*_ = -.10 (volatility × withdrawal).

### Secondary Analyses: Relationships between HPS Subscales and Personality Aspects

Previous research has suggested that the HPS may be usefully examined in terms of three subscales: social vitality, mood volatility, and excitement [40]. Conceptually, there may be some overlap between the HPS subscales and the personality aspects of extraversion and neuroticism. Specifically, the social vitality subscale of the HPS is similar to the assertiveness aspect of extraversion, excitement subscale of the HPS is similar to the enthusiasm aspect of extraversion, and the mood volatility subscale of the HPS is similar to the volatility aspect of neuroticism. We tested these links by constructing a series of general linear models predicting each personality aspect from positive affect, negative affect, and all three HPS subscales. Assertiveness (extraversion) was significantly related to social vitality (HPS), *β* = .48, but not mood volatility (HPS) or enthusiasm (HPS). Enthusiasm (extraversion) was related to all three HPS subscales: social vitality, *β* = .11, mood volatility, β = -.10, and excitement, *β* = .22. Volatility (neuroticism) was related to both social vitality (HPS), *β* = -.12, and mood volatility (HPS), *β* = .34. Finally, withdrawal (neuroticism) was related to all three HPS subscales: social vitality, *β* = -.25, mood volatility, *β* = .27, and excitement, *β* = -.08. Therefore, although there is some conceptual overlap between these scales, it does not appear that the full HPS can cleanly substitute for measuring these personality aspects.

## Discussion

The present research demonstrates that the personality traits of extraversion and neuroticism are significantly linked with subjective happiness and hypomania risk proneness, two affective outcomes shaped by the dispositional tendency to experience positive affect, above and beyond the role of affect alone. More importantly, we found that aspects of extraversion and neuroticism can usefully discriminate between subjective happiness and hypomania risk, providing greater nuance than when examining personality traits at their more general level and suggesting a link to the mechanism by which these affective outcomes are experienced. Subjective happiness was linked with high enthusiasm (extraversion) and low withdrawal (neuroticism). By contrast, hypomania risk was linked with high assertiveness (extraversion) and high volatility (neuroticism). That is, the ways extraversion and neuroticism manifest in subjective happiness and hypomania risk seem to be driven by different personality aspects: The happiness-extraversion relationship is driven by enthusiasm, whereas the hypomania risk-extraversion relationship is driven by assertiveness; the happiness-neuroticism relationship is driven by withdrawal, whereas the hypomania risk-neuroticism relationship is driven by volatility.

Consistent with the idea that assertiveness, enthusiasm, volatility, and withdrawal serve as different mechanisms for happiness and hypomania risk, our results suggest that these personality aspects interact to drive different kinds of behavior. With extraversion, happiness is highest when enthusiasm is high and assertiveness is low (and lowest when enthusiasm is low, regardless of assertiveness), and with neuroticism, happiness is highest when both volatility and withdrawal are low (and lowest when volatility is high). By contrast, hypomania risk is highest when assertiveness and enthusiasm are both high, and when volatility is high and withdrawal is low. These interactions demonstrate not only that extraversion and neuroticism are not unitary concepts in predicting behavior [24], but that types of extraversion and neuroticism can combine in varying ways to predict different types of trait affective outcomes.

Critically, these studies demonstrate that a single general construct–extraversion–manifests in different ways for subjective happiness and hypomania risk. This suggests that positive affect, which is shared by both outcomes, may be experienced within different psychological contexts depending on aspects of extraversion. That is, positive affect may not be experienced the same way for all individuals: rather, positivity may be shaped or interpreted in distinct ways depending on whether one is more predisposed to assertiveness or enthusiasm. These findings change our understanding of the meaning of positive affect in the context of subjective happiness. In contrast to positive affect serving as a general approach motivation, we suggest that subjective happiness is particularly facilitated by positivity that is shaped by an underlying orientation toward enthusiasm, denoting friendliness and sociability.

In addition to contributing toward understanding the divergent consequences of extraversion and positive affect, this work also highlights the divergent functional consequences of two types of neuroticism and negative affect. Previous research has suggested that negative affect can be characterized as relatively higher or lower in arousal [45]: high-arousal negative affect is related to anxiety, whereas low-arousal negative affect is related to avoidance. These characterizations of negative affect map nicely onto volatility and withdrawal. Though these strategies (anxiety and avoidance) are often complementary, the present work suggests that when decoupled they can have different implications. Hypomania risk was associated with greater volatility than withdrawal; functionally, this implies affective instability, which is probably related to difficulty disengaging in response to negative information. In addition, volatility and withdrawal are linked to different neurobiological systems that may underlie these orientations: volatility to the “fight-flight-freeze system” (FFFS) and withdrawal to the “behavioral inhibition system” (BIS) [46]. Whereas FFFS governs avoidant responses to immediate, inescapable threat, BIS governs anxiety and general vigilance for negative information.

The present work also helps to explain why a relationship between mania and neuroticism has not been consistently demonstrated in previous research. People experiencing manic episodes are often described as volatile and have difficulty inhibiting behavior when appropriate (i.e., high volatility / low withdrawal); this combination of aspects may be masked when examining neuroticism at the trait level. The possibility that one aspect is mainly responsible for the link between neuroticism and hypomania risk may explain why neuroticism and mania have not been consistently linked in the empirical literature despite their shared associated with negative affectivity. It makes sense that a measure of neuroticism that does not account for aspects would show no reliable relationship with mania. Indeed, recent work investigating personality aspects among people with bipolar disorder found that high volatility and low withdrawal predicts bipolar disorder over unipolar depression [47]. It would be interesting to investigate the relationships among volatility, withdrawal, and bipolar disorder when people are in a manic phase compared to a depressive phase to understand the influence of current symptoms on judgments of personality.

The clinical implications of this study are limited for two reasons. First, this study explored the relationship between hypomania risk and personality using a non-clinical sample in their dispositional levels of risk for hypomania. Second, we note that only a small number of participants in the present sample qualified as a clinically significant “high-risk” group (*n =* 84, or 8% of our sample) according to previously established cut-offs for the HPS (1.67 standard deviations above the mean, or a score of 34 in our sample) [37]. This may have made it more difficult to detect effects. As such, it remains to be determined precisely how these results would generalize to a clinical sample, and further research is warranted.

Future work investigating the relationship between positive affective outcomes and personality may explore the possibility that discrete positive emotional states interact with personality aspects in a different way than dispositional subjective happiness or hypomania risk. Although positive emotions have long been considered to be less specific than negative emotions, recent work has suggested that different positive emotions may have specific functions and consequences [48,49]. It would be interesting to explore the possibility that specific combinations of personality aspects facilitate the experience of specific positive emotional states which contribute to affective dispositions. Such work will also need to take into account the limitations of the PANAS [1,34] as a measurement tool for the full spectrum of positive affective states, and to contextualize PA more specifically as positive activation. A second direction for future work would be to investigate the varying functional consequences of additional personality aspects beyond extraversion and neuroticism. Openness, conscientiousness, and agreeableness also differentiate into aspects [23] and may have unique consequences for other outcomes. As the present work illustrates, it can be illuminating to take a more nuanced approach to considering personality in order to fully understand its diverse consequences for experience and behavior.

## Conclusions

The present work highlights the importance of leveraging insights from personality research to differentiate trait affective orientations. Although outcomes such as subjective happiness can be described by their affective qualities, it is also critical to characterize these outcomes by the personality mechanisms that shape affective experience. Positive affect by itself is insufficient for predicting how someone will interact with the world and cope with adversity. Our work suggests that for a deeper, richer understanding of subjective happiness–and possibly any affective disposition–the effects of positive and negative affect must be considered within the context of the whole person, including both psychological strengths and vulnerabilities.

## Supporting Information

S1 File Raw Data. Data for all waves can be accessed here.
(CSV)

S1 Table Results of Analyses Including Dropped Participants. We replicate the findings presented in the main document. Standardized beta values are presented.
(XLSX)

## References

[1] Watson D, Clark LA, Tellegen A (1988) Development and validation of brief measures of positive and negative affect: The PANAS scales. Journal of Personality and Social Psychology 54: 1063–1070. 3397865 10.1037//0022-3514.54.6.1063

[2] Fredrickson BL (1998) What good are positive emotions? Review of General Psychology 2: 300–319. 21850154 10.1037/1089-2680.2.3.300 PMC3156001

[3] Cacioppo JT, Gardner WL, Berntson GG (1997) Beyond bipolar conceputalizations and measures: The case of attitudes and evaluative space. Personality And Social Psychology Review 1: 3–25. 15647126 10.1207/s15327957pspr0101_2

[4] Diener E, Suh EM, Lucas RE, Smith HL (1999) Subjective well-being: Three decades of progress. Psychological Bulletin 125: 276–302.

[5] Ryan RM, Deci EL (2001) On happiness and human potentials: A review of research on hedonic and eudaimonic well-being. Annual Review of Psychology 52: 141–166. 11148302 10.1146/annurev.psych.52.1.141

[6] Cohn MA, Fredrickson BL, Brown SL, Mikels JA, Conway AM (2009) Happiness unpacked: Positive emotions increase life satisfaction by building resilience. Emotion 9: 361–368. 10.1037/a0015952 19485613 PMC3126102

[7] Diener E, Seligman MEP (2002) Very happy people. Psychological Science 13: 81–84. 11894851 10.1111/1467-9280.00415

[8] Diener E, Chan MY (2011) Happy people live longer: Subjective well-being contributes to health and longevity. Applied Psychology: Health and Well-Being 3: 1–43.

[9] Lyubomirsky S, King L, Diener E (2005) The benefits of frequent positive affect: Does happiness lead to success? Psychological Bulletin 131: 803–855. 16351326 10.1037/0033-2909.131.6.803

[10] Fredrickson BL, Joiner T (2002) Positive emotions trigger upward spirals toward emotional well-being. Psychological Science 13: 172–175. 11934003 10.1111/1467-9280.00431

[11] Diener E, Sandvik E, Pavot W (2009) Happiness is the frequency, not the intensity, of positive versus negative affect In: Diener E, editor. Assessing Well-Being: The Collected Works of Ed Diener: Springer pp. 213–231.

[12] Gruber J, Mauss IB, Tamir M (2011) A dark side of subjective happiness? How, when, and why subjective happiness is not always good. Perspectives on Psychological Science 6: 222–233. 26168514 10.1177/1745691611406927

[13] Johnson SL (2005) Mania and dysregulation in goal pursuit: A review. Clinical Psychology Review 25: 241–262. 15642648 10.1016/j.cpr.2004.11.002 PMC2847498

[14] Eckblad M, Chapman LJ (1986) Development and validation of a scale for hypomanic personality. Journal of Abnormal Psychology 95: 214–222. 3745642 10.1037//0021-843x.95.3.214

[15] Meyer B, Bauer M (2009) Positive and negative affect in individuals at high and low risk for bipolar disorders. Journal of Individual Differences 30: 169–175.

[16] Hofmann BU, Meyer TD (2006) Mood fluctuations in people putatively at risk for bipolar disorders. British Journal of Clinical Psychology 45: 105–110. 16480569 10.1348/014466505X35317

[17] Gruber J, Johnson SL, Oveis C, Keltner D (2008) Risk for mania and positive emotional responding: Too much of a good thing? Emotion 8: 23–33. 10.1037/1528-3542.8.1.23 18266513 PMC2847501

[18] Costa PT, McCrae RR (1980) Influence of extraversion and neuroticism on subjective well-being: Happy and unhappy people. Journal of Personality and Social Psychology 38: 668–678. 7381680 10.1037//0022-3514.38.4.668

[19] Gruber J, Devlin HC, Moskowitz J. (2014) Seeing both sides: An introduction to the light and dark sides of positive emotion In: Gruber J & Moskowitz J, editors. Positive Emotion: Integrating the Light Sides and the Dark Sides. New York: Oxford University Press.

[20] Clark LA, Watson D (2008) Temperament: An organizing paradigm for trait psychology In: John OP, Robins RW, Pervin LA, editors. Handbook of personality: Theory and research. 3 ed. New York: Guilford Press pp. 265–286.

[21] Depue RA, Collins PF (1999) Neurobiology of the structure of personality: Dopamine, facilitation of incentive motivation, and extraversion. Behavioral and Brain Sciences 22: 491–569. 11301519 10.1017/s0140525x99002046

[22] Costa PT, McCrae RR (1992) The five-factor model of personality and its relevance to personality disorders. Journal of Personality Disorders 6: 343–359.

[23] DeYoung CG, Quilty LC, Peterson JB (2007) Between facets and domains: 10 aspects of the Big Five. Journal of Personality and Social Psychology 93: 880–896. 17983306 10.1037/0022-3514.93.5.880

[24] Jang KL, Livesley WJ, Angleitner A, Riemann R, Vernon PA (2002) Genetic and environmental influences on the covariance of facets defining the domains of the five-factor model of personality. Personality and Individual Differences 33: 83–101.

[25] Berridge KC, Kringelbach ML (2008) Affective neuroscience of pleasure: Reward in humans and animals. Psychopharmacology 199: 457–480. 10.1007/s00213-008-1099-6 18311558 PMC3004012

[26] Berridge KC, Robinson TE, Aldridge JW (2009) Dissecting components of reward: ‘Liking’, ‘wanting’, and learning. Current Opinion in Pharmacology 9: 65–73. 10.1016/j.coph.2008.12.014 19162544 PMC2756052

[27] Berridge KC, Robinson TE (2003) Parsing reward. Trends in Neurosciences 26: 507–513. 12948663 10.1016/S0166-2236(03)00233-9

[28] Meyer B, Johnson SL, Winters R (2001) Responsiveness to threat and incentive in bipolar disorder: Relations of the BIS/BAS scales with symptoms. Journal of Psychopathology and Behavioral Assessment 23: 133–143. 21765592 10.1023/A:1010929402770 PMC3137128

[29] Gruber J (2011) Can feeling too good be bad? Positive emotion persistence (PEP) in bipolar disorder. Current Directions in Psychological Science 20: 217–221.

[30] Carpenter S (2012) Psychology's bold initiative. Science 335: 1558–1561. 10.1126/science.335.6076.1558 22461583

[31] Schimmack U (2012) The ironic effect of significant results on the credibility of multiple-study articles. Psychological Methods 17: 551–566. 10.1037/a0029487 22924598

[32] Buhrmester M, Kwang T, Gosling SD (2011) Amazon's Mechanical Turk: A new source of inexpensive, yet high-quality, data? Perspectives on Psychological Science 6: 3–5. 26162106 10.1177/1745691610393980

[33] Cohen J (1988) Statistical Power Analysis for the Behavioral Sciences. 2 ed. Hillsdale, NJ: Erlbaum.

[34] Watson D, Wiese D, Vaidya J, Tellegen A (1999) The two general activation systems of affect: Structural findings, evolutionary considerations, and psychobiological evidence. Journal of Personality and Social Psychology 76: 820–830.

[35] Lyubomirsky S, Lepper HS (1999) A measure of subjective happiness: Preliminary reliability and construct validation. Social Indicators Research 46: 137–155.

[36] Klein DN, Lewinsohn PM, Seeley JR (1996) Hypomanic personality traits in a community sample of adolescents. Journal of Affective Disorders 38: 135–143. 8791182 10.1016/0165-0327(96)00005-5

[37] Kwapil TR, Miller MB, Zinser MC, Chapman LJ, Chapman J, Eckblad M (2000) A longitudinal study of high scorers on the Hypomanic Personality Scale. Journal of Abnormal Psychology 109: 222–226. 10895560

[38] Andrews FM, Withey SB (1976) Social indicators of well-being New York: Plenum.

[39] Diener E, Emmons RA, Larsen RJ, Griffin S (1985) The satisfaction with life scale. Journal of Personality Assessment 49: 71–75. 16367493 10.1207/s15327752jpa4901_13

[40] Schalet BD, Durbin CE, Revelle W (2011) Multidimensional structure of the Hypomanic Personality Scale. Psychological Assessment 23: 504–522. 10.1037/a0022301 21480726

[41] Freedman J (1978) Happy people: What happiness is, who has it, and why New York: Harcourt Brace Jovanovich.

[42] Altman EG, Hedeker D, Peterson JL, Davis JM (1997) The Altman Self-Rating Mania Scale. Society of Biological Psychiatry 42: 948–955. 10.1016/S0006-3223(96)00548-3 9359982

[43] Beck AT, Rial WY, Rickels K (1974) Short form of Depression Inventory: Cross-validation. Psychological Reports 34: 1184–1186. 4424377

[44] Parker G, Fletcher K, McCraw S, Hong M (2014). The hypomanic personality scale: A measure of personality and/or bipolar symptoms? Psychiatry Research 220: 654–658. 10.1016/j.psychres.2014.07.040 25156658

[45] Russell JA (1980) A circumplex model of affect. Journal of Personality and Social Psychology 39: 1161–1178. 10.1037//0022-3514.79.2.286 10948981

[46] Gray JA, McNaughton N (2000) The neuropsychology of anxiety, 2nd edition New York: Oxford University Press.

[47] Quilty LC, Pelletier M, DeYoung CG, Bagby RM (2013). Hierarchical personality traits and the distinction between unipolar and bipolar disorders. Journal of Affective Disorders 147: 247–254. 10.1016/j.jad.2012.11.012 23261133

[48] Campos B, Shiota MN, Keltner D, Gonzaga GC, Goetz JL (2013) What is shared, what is different? Core relational themes and expressive displays of eight positive emotions. Cognition & Emotion 27: 37–52. 22716231 10.1080/02699931.2012.683852

[49] Shiota MN, Keltner D, John OP (2006) Positive emotion dispositions differentially associated with Big Five personality and attachment style. The Journal of Positive Psychology 1: 61–71.

